# Penile cancer: a rare clinical image

**DOI:** 10.11604/pamj.2025.52.170.46407

**Published:** 2025-12-18

**Authors:** Switi Jawade, Pratibha Wankhede

**Affiliations:** 1 Department of Obstetrics and Gynecology Nursing, Shalinitai Meghe College of Nursing, Salod (Hirapur), Datta Meghe Institute of Higher Education and Research (Deemed to be University), Sawangi, Wardha, Maharashtra, India,; 2 Department of Community Health Nursing, Shalinitai Meghe College of Nursing, Salod (Hirapur), Datta Meghe Institute of Higher Education and Research (Deemed to be University), Sawangi, Wardha, Maharashtra, India

**Keywords:** Penile cancer, squamous-cell carcinoma, urological cancer

## Image in medicine

Penile cancer is a very rare type of urological cancer that mostly occurs in the epithelium of the inner glans. Its major etiological factors are phimosis, smoking, and poor penile hygiene. The disease extends gradually and affects the complete glans penis and the shaft of the penis. It mostly occurs in men aged between 40 and 70 years. With early diagnosis and treatment, further complications can be prevented. We here report the case of a 52-year-old patient who was admitted to the male surgery ward with no significant past medical history with the complaint of swelling over the tip of the penis associated with throbbing pain over the shaft of the penis. He was apparently alright 10 years back when he first noticed a small mass over the penile shaft, which was of insidious onset and progressive in nature. Initially, the mass was 0.5 x 0.5cm in size, which gradually increased to the current size of 3 x 2cm. Over time, the lesion extended and became painful. Clinical examination revealed a progressive penile mass involving the glans and shaft of the penis, with bilateral inguinal lymphadenopathy. Fine needle aspiration cytology (FNAC) was performed from both inguinal lymph. A routine blood investigation was also carried out. Fine needle aspiration cytology findings confirmed bilateral inguinal lymph node metastasis, suggestive of a malignant epithelial tumor. Based on clinical findings and cytological examination, the final diagnosis was well-differentiated squamous cell carcinoma of the penis with bilateral inguinal lymph node metastasis. The patient was referred to the surgery unit for further oncological evaluation and definitive management. At the time of reporting, the patient had been referred for specialized surgical management; short and medium-term outcomes were not available at the time of this report.

**Figure 1:**
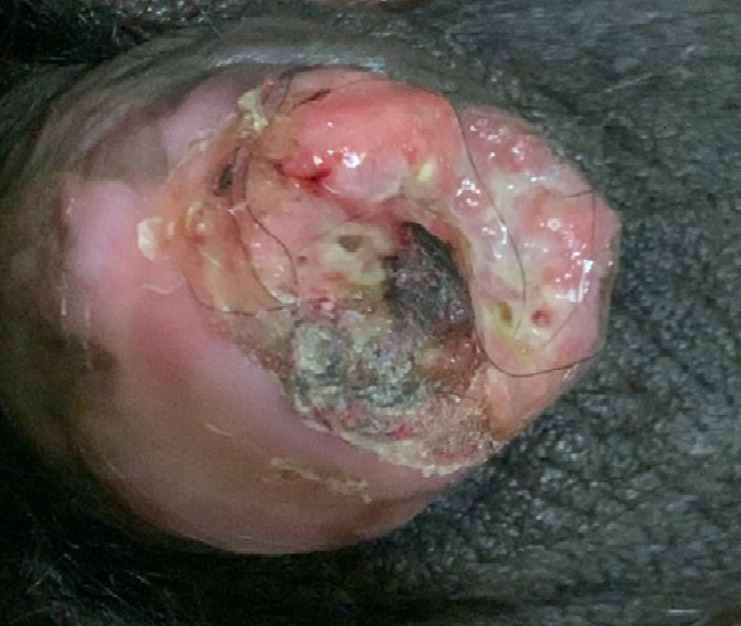
appearance of penile cancer (invasion of glans penis)

